# OME-NGFF: a next-generation file format for expanding bioimaging data access strategies

**DOI:** 10.1038/s41592-021-01326-w

**Published:** 2021-11-29

**Authors:** Josh Moore, Chris Allan, Sébastien Besson, Jean-Marie Burel, Erin Diel, David Gault, Kevin Kozlowski, Dominik Lindner, Melissa Linkert, Trevor Manz, Will Moore, Constantin Pape, Christian Tischer, Jason R. Swedlow

**Affiliations:** 1 University of Dundee, Dundee, Scotland, UK; 2 Glencoe Software, Inc. Seattle, WA, USA; 3 Harvard Medical School, Boston, MA, USA; 4 European Molecular Biology Laboratory (EMBL), Heidelberg, Germany

## Abstract

The rapid pace of innovation in biological imaging and the diversity of its applications have prevented the establishment of a community-agreed standardized data format. We propose that complementing established open formats like OME-TIFF and HDF5 with a next generation file format like Zarr will satisfy the majority of use cases in bioimaging. Critically, a common metadata format used in all these vessels can deliver truly findable, accessible, interoperable and reusable bioimaging data.

Biological imaging is one of the most innovative fields in the modern biological sciences. New imaging modalities, probes, and analysis tools appear every few months and often prove decisive for enabling new directions in scientific discovery. One feature of this dynamic field is the need to capture new types of data and data structures. While there is a strong drive to make scientific data Findable, Accessible, Interoperable and Reusable (FAIR ^1^), the rapid rate of innovation in imaging has resulted in the creation of hundreds of proprietary file formats (PFFs) and has prevented the unification and adoption of standardized data formats. Despite this, the opportunities for sharing and integrating bioimaging data and, in particular, linking these data to other "omics" datasets have never been greater. Therefore, to every extent possible, increasing "FAIRness" of bioimaging data is critical for maximizing scientific value, as well as for promoting openness and integrity ^2^.

When working with a large number of PFFs, interoperability and accessibility are achieved using translation and conversion provided by open source, community-maintained libraries that produce an open, common data representation. On-the-fly translation produces a transient representation of bioimage metadata and binary data in an open format but must be repeated on each use. In contrast, conversion produces a permanent copy of the data, again in an open format, bypassing bottlenecks in repeated data access. As workflows and data resources emerge that handle terabytes (TB) to petabytes (PB) of data, the costs of on-the-fly translation have become bottlenecks to scientific analysis and the sharing of results. Open formats like OME-TIFF ^3^ and HDF5 ^4^ are often used for permanent conversion, but both have limitations that make them ill-suited for use cases that depend on very high and frequent levels of access, e.g., training of AI models and publication of reference bioimage datasets in cloud-based resources. For these situations, the community is missing a multidimensional, multiresolution binary container that provides parallel read and write capability, that is natively accessible from the cloud (i.e., without server infrastructure), and that has a flexible, comprehensive metadata structure (see Supplementary Note for more details).

To this end, we have begun building OME’s next-generation file format (OME-NGFF) as a complement to OME-TIFF and HDF5. Together these formats provide a flexible set of choices for bioimaging data storage and access at scale over the next decade and, potentially, a common, FAIR solution for all members of the biological imaging community -- academic and industrial researchers and imaging scientists, and academic and commercial technology developers.

## Next-generation file formats

We use the term “Next-generation file formats” (NGFFs) to denote file formats which can be hosted natively in an object (or “cloud”) storage for direct access by a large number of users. Our current work, which we refer to as OME-NGFF, is built upon the Zarr format ^5^ but heavily informed and connected to both TIFF and HDF5. We have compared the characteristics of these three open formats in Supplementary Table 1.

To date, the development of OME-NGFF has focused on pixel data and metadata specifications for multidimensional, multiscale images, high-content screening datasets, and derived labelled images. These specifications include support for “chunking”, or storage of parts of the binary pixel data in smaller files that support rapid access to the data from orthogonal views or different resolution levels (also known as “pyramidal data”). Labeled images, such as segmentation or classification masks can now remain in a common data structure with the original pixel data and metadata, providing a single mechanism for tracking the provenance of original and derived data allowing programmatic rather than manual management.

**Extended Data Fig. 1:**
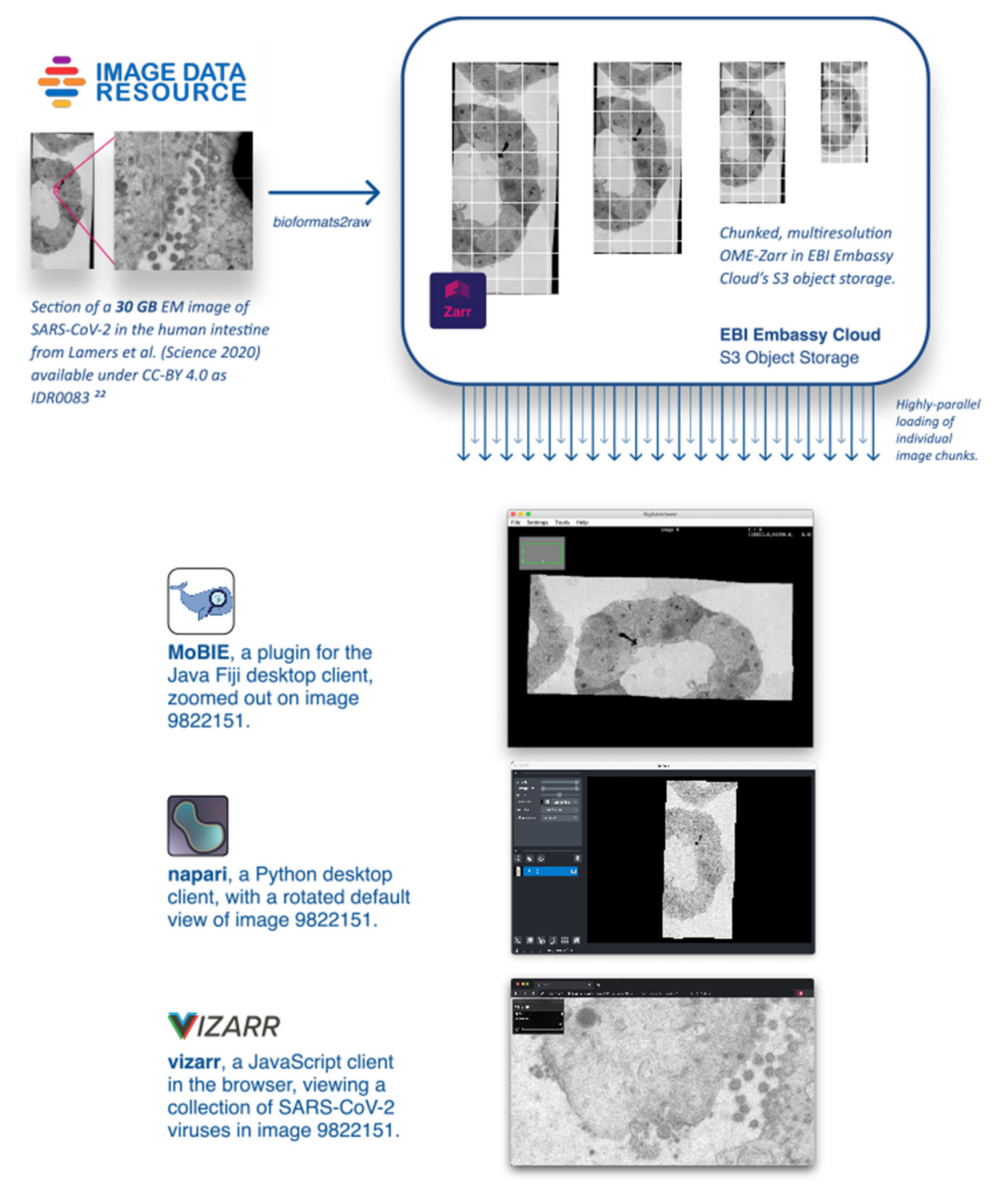
Maximizing re-use by allowing popular tools to access bioimaging data in the cloud. An example of using NGFFs for promoting the distribution of public image datasets. Selection of current tools streaming different portions of the same SARS-CoV-2 virus image at various resolutions directly from S3 storage at the European Bioinformatics Institute (EBI). Original data from Lamers et al. is available in IDR while the converted data is available on Zenodo.21-23.

We have also built multiple implementations of these specifications, demonstrating the usability and performance of these formats. *bioformats2raw* can be used for writing OME-NGFF from standalone Java applications and *omero-cli-zarr* is available for exporting from OMERO ^6^. Reading is implemented in *ome-zarr-py* which has been integrated into the *napari* viewer ^7^, in *Fiji* via the *MoBIE* plugin ^8^, and finally via Viv-based *vizarr* for access in the browser ^9^. Permissively-licensed example datasets from the Image Data Resource (IDR) ^10^ have been converted into Zarr and stored in an S3 object storage bucket for public consumption (Extended Data Figure 1). Though OME-NGFF is still in development, each of these implementations is an example of how data access and application is simplified by having a universal data storage pattern. Current and future specifications are published under https://ngff.openmicroscopy.org/latest/.

## Bioimage Latency Benchmark

To demonstrate how NGFFs complement available, open formats, we have built and published a benchmark -- *bioimage-latency-benchmark* -- that compares random, serial access speeds to uncompressed TIFF, HDF5, and Zarr files. These measurements provide an upper bound on the overhead that a user would experience accessing the formats using common libraries, tifffile, h5py and zarr-python respectively. Though future extensions to the benchmark are intended, we have focused on a single, serverless Python environment since one library -- *fsspec* -- can be used to access all three data formats across multiple storage mechanisms without the need for any additional infrastructure.

**Figure 1:**
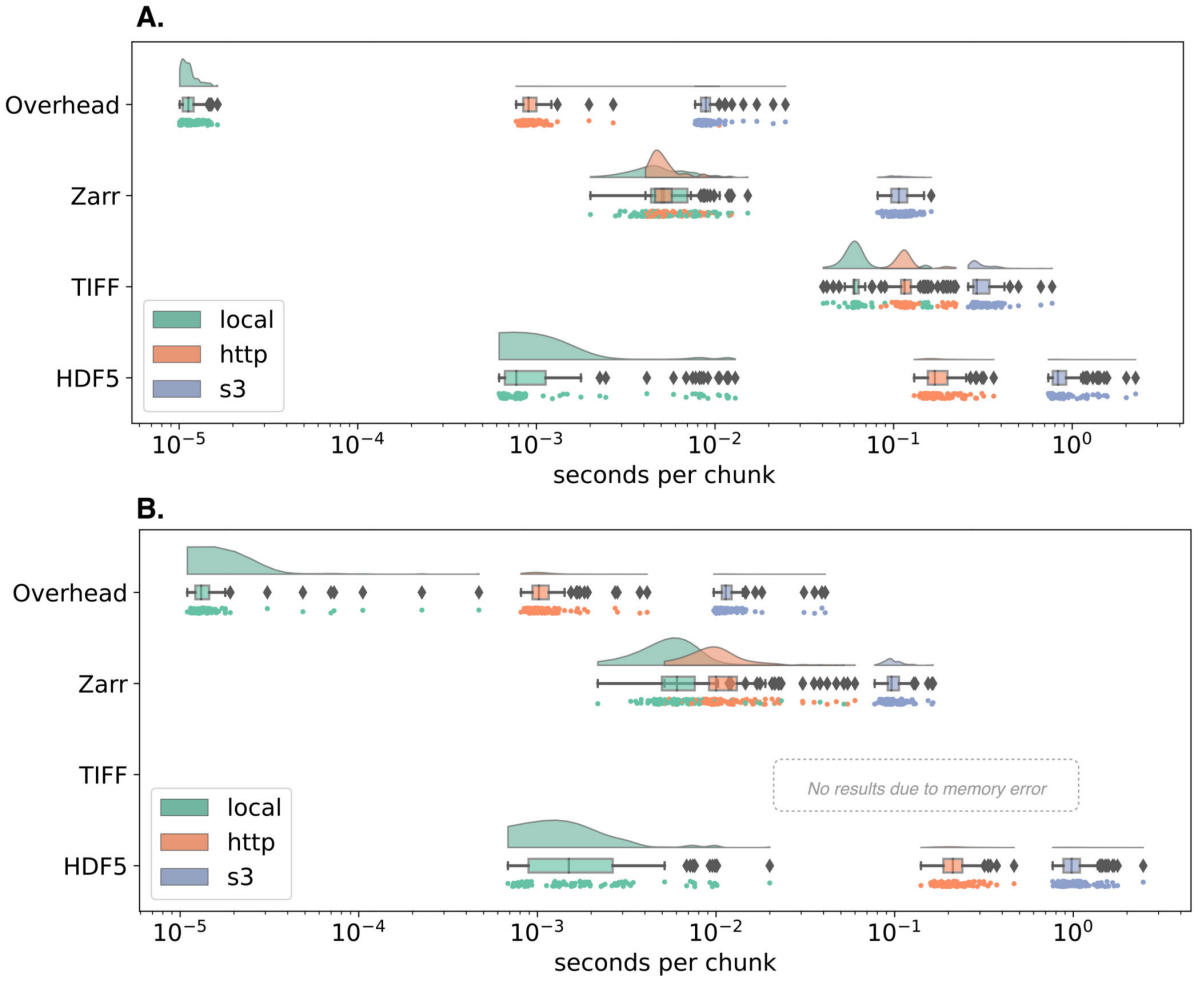
Chunk retrieval time is less sensitive to data location with next-generation file formats. Random sampling of 100 chunks from synthetically generated, 5D images measures access times for three different formats on the same file system ("local", green), over HTTP using the nginx web server ("http", orange), and using Amazon’s proprietary S3 object storage protocol ("s3", blue) under two scenarios: (A) a whole-slide CycIF imaging dataset with many large planes of data (x=64k, y=64k, c=8) and chunks of 256×256 pixels (128 KB) and (B) a time-lapse LSM dataset with isotropic dimensions (x=1024, y=1024, z=1024, t=100) and chunks of 32×32×32 pixels (64 KB). See the Methods for more information.

The benchmark includes instructions for running on Docker or AWS EC2 and contains all necessary code to regenerate representative samples for two established imaging modalities: large multi-channel two-dimensional images like the ones produced by cyclic immunofluorescence (CycIF) ^11^ and timelapse isotropic volumes typically generated by LSM ^12^. Each synthetic HDF5, TIFF and Zarr dataset was generated by first invoking the ImarisWriter, then converting the HDF5-based Imaris files into Zarr with *bioformats2raw*, and finally converting the Zarr to TIFF with *raw2ometiff*. All three datasets along with a 1-byte dummy file for measuring overhead were placed in three types of storage: local disk, a remote server, and object storage. We measured the reading time of individual chunks for all four file types across the three storage systems. Figure 1 shows that as the latency of access grows, access times for monolithic formats like TIFF and HDF5 increase because libraries must seek the appropriate data chunk, whereas NGFF formats like Zarr provide direct access to individual chunks. In the 3D case, the TIFF data was too large to fit into local memory and the benchmark errored.

On local storage, access speeds for NGFF files were similar to HDF5 and both substantially outperformed TIFF. This matches previous results showing that a number of factors must be taken into account to determine the relative performance of HDF5 and Zarr ^13^. Together these results partially explain HDF5’s popularity for desktop analysis and visualization of LSM datasets.

However, on cloud storage, access speeds for NGFF files are at least an order of magnitude faster than HDF5. Parallel reads ^14^, supporting streaming of image data files from remote http-based or cloud-based servers give performance similar to local disk access. Data streaming obviates the need for wholesale data download and is especially important for providing performant access to multi-TB datasets.

**Extended Data Fig. 2:**
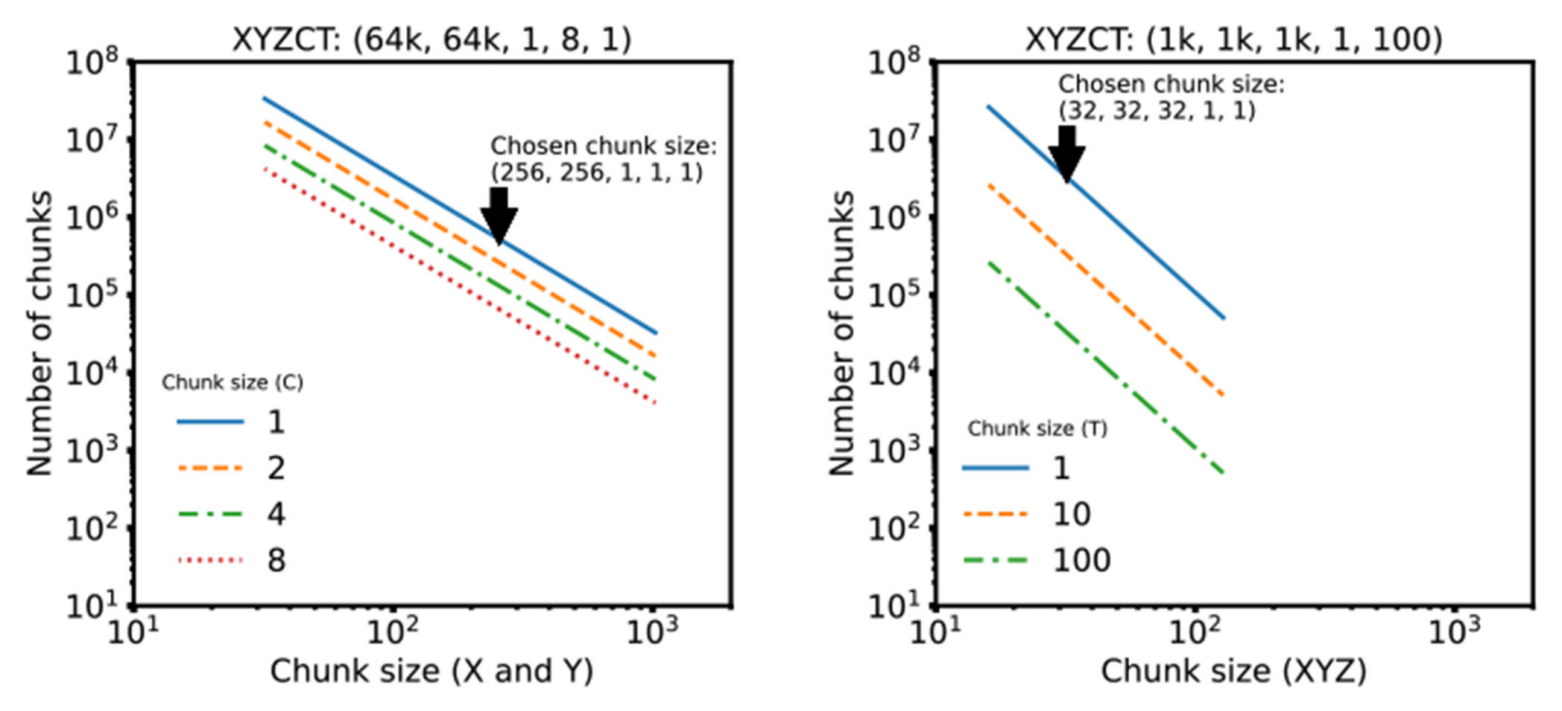
Effect of Chunk Size on Chunk Number For each modality, the chunk size of the benchmark dataset was chosen as the best compromise between the size of individual chunks and the total number of chunks in the Zarr dataset. The plots above show typical power of 2 chunk sizes: between 32 and 1024 for the 2D data and between 16 and 128 for the 3D data. We chose a 2D chunk size of 256×256 for the CyIF-like dataset and a 3D chunk size of 32×32×32 for the LSM-like dataset. Note that due to the planar limitation of TIFF, the LSM dataset was stored as 2D TIFF tiles of size 32×32 but the benchmark looped over 32 tiles to measure the access time of the same chunk size.

We note that our benchmark measures direct access to underlying storage. Additional applications, e.g., HSDS for HDF5 or OMERO for TIFF, may improve the performance of specific use cases, but add significant complexity to any deployment and make direct comparisons between the different data access regimes in Figure 1 difficult. Additionally, a key parameter in overall access times is the size of individual chunks. As chunk sizes decrease, the number of individual chunk files increases rapidly (See Extended Data Figure 2). In this benchmark, we have chosen a compromise between chunk size and number of individual files. This illustrates a primary downside of NGFF formats: as the number of files increases, the time required for copying data between locations increases. Users will need to understand and balance these trade-offs when choosing between open, bioimaging file formats.

## Outlook: Community Adoption

We assert that together low-latency, cloud-capable NGFF, TIFF and HDF5 can provide a balanced set of options that the community can converge upon, and slow the development of ever more file formats. To this end, OME is committed to building an interoperable metadata representation across all three file formats to ensure ease of adoption and data exchange (see Supplementary Note for more information).

When data is frequently accessed, e.g., as a public resource or a training dataset, upfront conversion will lead to overall time savings. In situations where object storage is mandated as in large scale public repositories, we encourage the use of OME-NGFF today. Alternatively, users needing to transfer their images may choose to store their data in a large single file like HDF5. OME-TIFF remains a safe option for those who rely on proprietary software for visualization and analysis, especially in digital pathology and other whole slide image applications, as many have been extended to both read and write this open standard. Each choice comes with benefits and costs, and individual scientists, institutions, global collaborations and public data resources need the flexibility to decide which approach is suitable. We encourage the community to choose from the most appropriate of the formats described above, secure in the knowledge that conversion is possible if it becomes necessary.

We foresee this being a critical strategy where data generated in advanced bioimaging applications is converted into an optimized format for downstream processing, analysis, visualization and sharing. All subsequent data access occurs via open data formats without the need for repeated, on-the-fly translation. We have begun implementing this workflow in the IDR (Extended Data Figure 1), alleviating the need for time consuming downloads and cross-referencing metadata and resulting in substantially more accessible and interoperable data. We look forward to working with other resources to further develop this policy. Further, as adoption of public image data resources increases, commercial vendors will hopefully engage with these efforts to support their customers, who are increasingly required to publish datasets as supplementary material. Moreover, some commercial imaging companies are themselves building cloud-based data handling and analysis solutions (e.g., https://www.apeer.com), thus broadening the community of users who need cloud-competent file formats.

Ultimately, we hope to see digital imaging systems producing open, transparent, in other words FAIR, data without the need for further conversion. Until that time, we are committed to providing the data conversion needs of the community. Following the same pattern established by *bioformats2raw* and *raw2ometiff*, we propose to meet this challenge via a set of migration tools allowing efficient data transformations between all data formats contained in this suite of interoperable formats. Additionally, as the specification evolves based on community feedback, the same migration tools will allow upgrading the scientific data generated by the bioimaging community to prevent the need for long-term maintenance of older data. Upcoming specifications include geometric descriptions of regions of interest, meshes, and transformations for correlative microscopy.

To provide the best chance of wide adoption and engagement, we are developing the formats in the open, with frequent public announcements of progress and releases of reference software and examples (https://forum.image.sc/tag/ome-ngff) and regular community meetings where we present work, source feedback, and encourage community members, including vendors, to participate in the specification and implementation. The community process is being developed and we welcome contributions from all interested parties on https://github.com/ome/ngff.

## Methods

### Bioimage latency benchmark: synthetic data generation

#### Imaging modality and dataset sizes

Synthetic datasets were generated for two established imaging modalities: a large multi-channel two-dimensional image typical of cyclic immunofluorescence (CycIF) ^11^ of XYZCT dimensions 64000×64000×1×8×1 and a timelapse isotropic volume typical of LSM ^12^ of XYZCT dimensions 1024×1024×1024×1×100.

For each modality, the chunk size of the benchmark dataset was chosen as the best compromise between the size of individual chunks and the total number of chunks in the Zarr dataset. To make this decision, the individual chunk size was computed against the total number of chunks for typical sizes ranging from 16 up to 1024 (see chunks.py^15^ and Extended Data Figure 2). Based on this, we chose a 2D chunk size of 256×256 for the CycIF-like dataset and a 3D chunk size of 32×32×32 for the LSM-like dataset. Note that due to the planar limitation of TIFF, the LSM dataset was stored as 2D TIFF tiles of size 32×32 but the benchmark loaded 32 tiles to measure the total access time.

All data was stored uncompressed to keep chunk sizes consistent for the random generated data. Note that with the default aws s3 cp command, data upload decreased from over 100MiB/s for the single HDF5 file to under 20MiB/s for the Zarr dataset.

#### Dataset generation

The HDF5 version of each synthetic dataset was first generated by using the ImarisWriter library ^16^ (version 2021-04-07) with a version of the ImarisWriterTest example ^16,17^ modified to allow setting the desired chunk size and generate gradient images rather than random data. This HDF5-based Imaris file was converted into Zarr using a modified version of *bioformats2raw* 0.2.6 with support for chunks using a “/” dimension separator ^18^. Finally the Zarr was converted into TIFF with a modified version of *raw2ometiff* 0.2.6 allowing it to consume Zarr filesets with a “/” dimension separator ^19^. Both modifications have been released since in *bioformats2raw* 0.3.0 and *raw2ometiff 0*.3.0.

For the CycIF-like dataset, this conversion generated a single 86G TIFF file, a single 86G HDF5 file and a Zarr dataset composed of 700k files of 86G in total. For the LSM-like dataset, the conversion generated a single 300G TIFF file, a single 229G HDF5 file and a Zarr dataset of 4.3M files of 264G in total.

### Bioimage latency benchmark: measurements and results

#### Measurements

All three datasets along with a 1-byte dummy file for measuring overhead were placed in three types of storage: local disk, a remote server, and object storage. We measured the reading time of individual chunks for all four file types across the three storage systems.

A random sequence of 100 chunk locations was chosen for the benchmark. All 100 chunks were loaded from each file in the same order. The time taken to retrieve the chunk, independent of the time taken to open a file or prepare the remote connection, was recorded.

#### Raincloud plots

Raincloud plots ^20^ combine three representations (split-half violin plots, box plots, raw data points) so that the true distribution and the statistical parameters can be compared. Split-half violin plots show a smoothed version of a histogram with a kernel density estimate (KDE). This type of plot is useful to determine, at a glance, if the mean is lower or higher than the median depending on the skewness of the curve. Box plots show the median and the boundaries of quartiles on either side of the median of the distribution to determine statistical differences at a glance. Below each box plot, the raw data points are additionally plotted with slight vertical jittering to avoid overlaps.

All code for reproducing the plots and the runs both locally with Docker or Amazon EC2 instances are available under a BSD-2 license on Zenodo ^15^.

## 1 Extended Data

**Extended Data Fig. 3:**
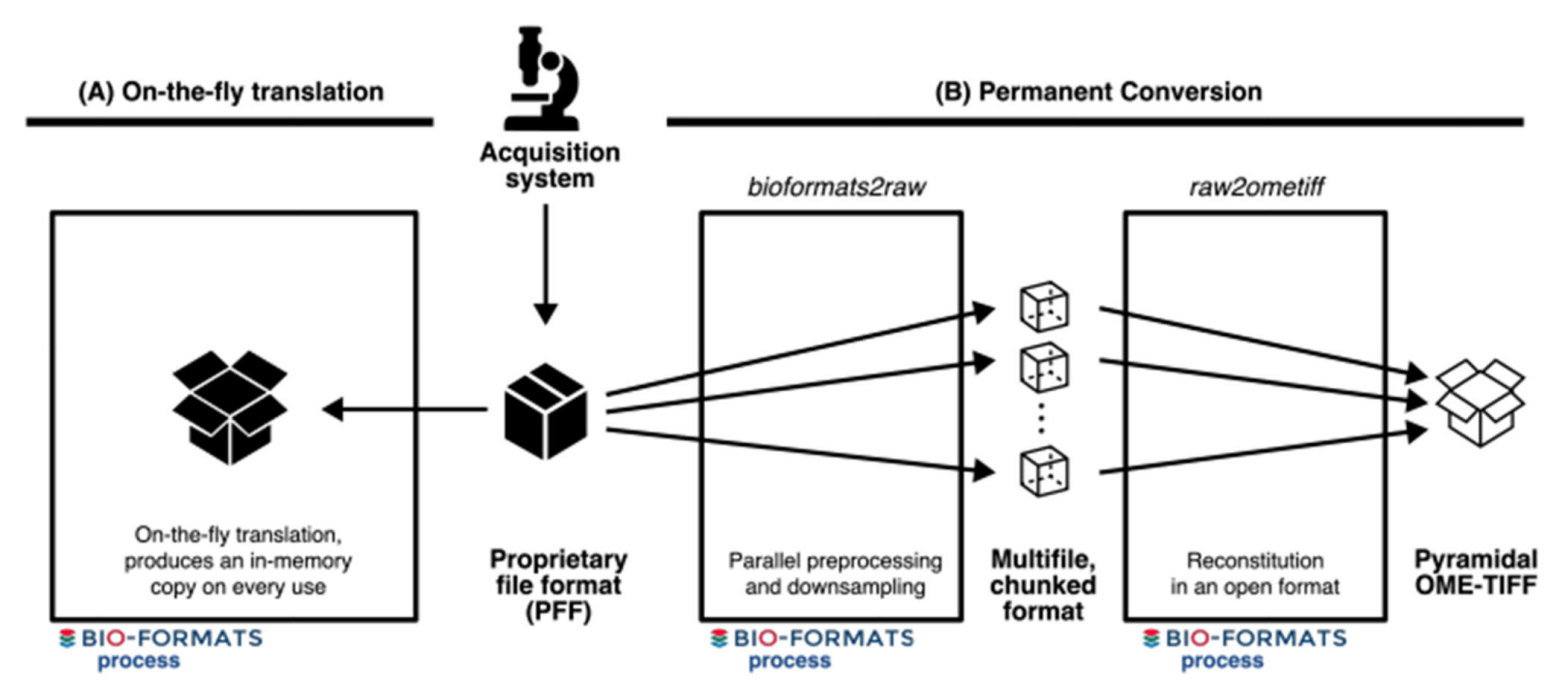
Conversion tools provide an alternative to continual, on-the-fly translation of PFFs. Figure shows workflows for file format access. (A) The classical approach to access images produced by an acquisition system is to use a library like Bio-Formats to translate the proprietary file format (PFF) and produce an in-memory copy of the imaging data on-the-fly. This translation needs to be repeated on every use. (B) With the existence of open, community-supported formats, converting PFFs becomes the most cost-efficient method for long-term storage and sharing of microscopy data. bioformats2raw and raw2ometiff, described below, parallelize the creation of an open format, OME-TIFF, by using an intermediate format consisting of many, individual files each with one chunk of the original image data.

**Extended Data Fig. 4:**
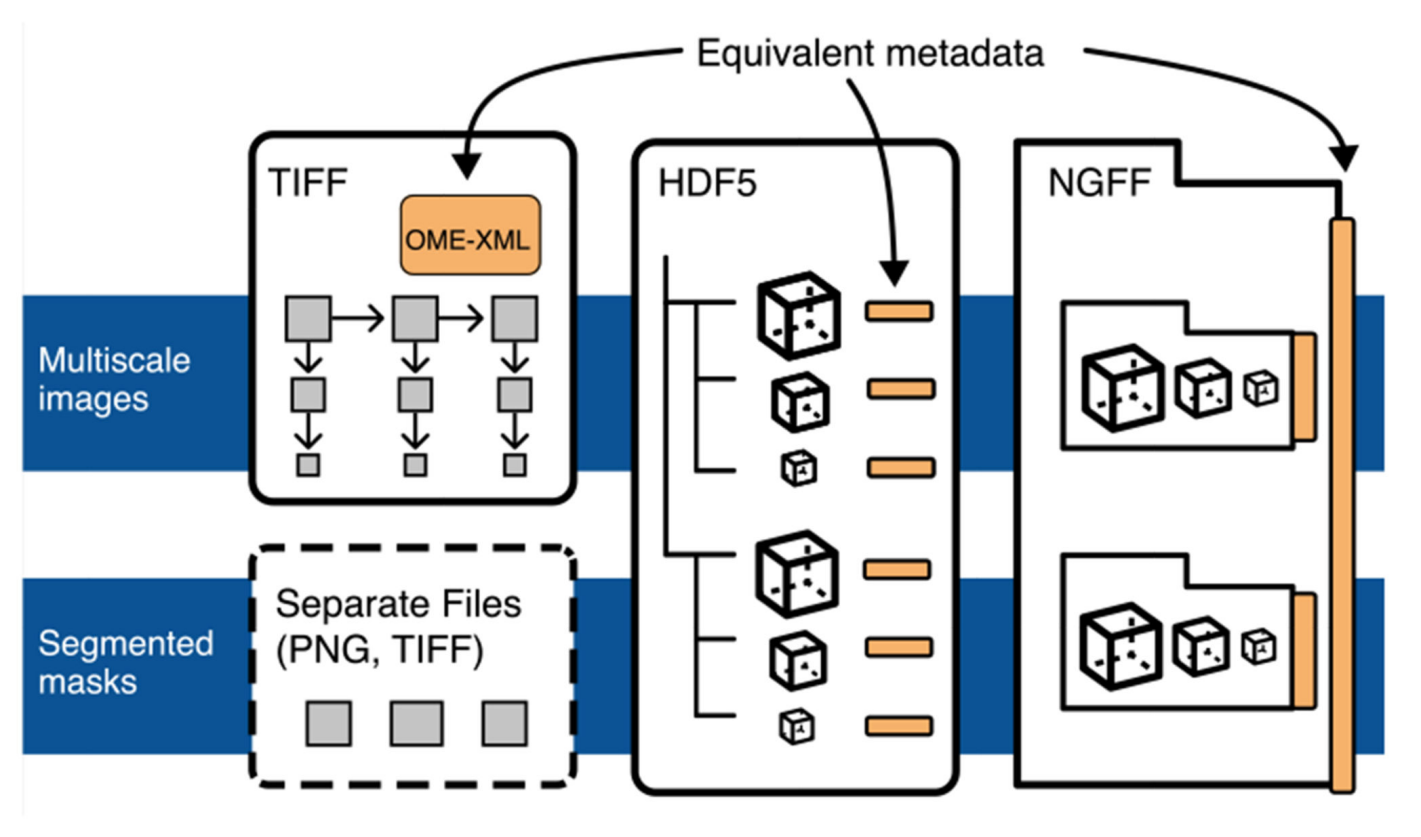
Unification of metadata specifications will allow interoperability between TIFF, HDF5, and Zarr. Each proposed container (TIFF, Zarr, HDF5) can be used interchangeably to store pixel data, but trade-offs described in this manuscript can be used to determine what is the best target. TIFF is ideal for interoperability in digital pathology and other 2-dimensional domains since the format is widely accessible by established open source and proprietary software. In higher-dimensional domains, HDF5 and Zarr are better suited. HDF5 will likely be preferred for local access. If data is intended for sharing in the cloud, Zarr will likely be preferred. High throughput image analysis will benefit from the lower-latency access to data in HDF5 and Zarr. If original image data is paired with derived representations like pixel or object classification, a shared structure in HDF5 or Zarr is likely the best choice.

## Supplementary Material

Supplementary Information

## Data Availability

The synthetic data generated for the benchmark is 1.05 TB. All code necessary to regenerate the data, including at different sizes, is available on Zenodo under a BSD-2 license ^15^. The SARS-CoV-2 EM dataset from Extended Data Figure 1, originally from Lamers et al. ^21^ and published in IDR ^22^, was converted into OME-NGFF and is available at Zenodo 23 under a CC-BY 4.0 license.

## Editor’s summary

OME’s next-generation file format (OME-NGFF) provides a cloud-native complement to OME-TIFF and HDF5 for storing and accessing bioimaging data at scale, and works toward the goal of findable, accessible, interoperable and reusable bioimaging data.

## Code Availability

Data generation and analysis code for file format benchmarking is available on Zenodo under a BSD-2 license ^15^.
