# Supplementary material for: OME-NGFF: a next-generation file format for expanding bioimaging data-access strategies
Source: Nat Methods. 2021 Nov 29;18(12):1496–8. doi: 10.1038/s41592-021-01326-w (PMC8648559; doi:10.1038/s41592-021-01326-w)
Supplement: Supplementary file 1 [file 41592_2021_1326_MOESM1_ESM.pdf]

nature portfolio

https://doi.org/10.1038/s41592-021-01326-w

Supplementary information

OME-NGFF: a next-generation file format for expanding bioimaging data-access strategies

In the format provided by the authors and unedited

Supplementary Information

OME-NGFF: a next-generation file format for expanding bioimaging data access strategies

Josh Moore[1], Chris Allan[2], Sébastien Besson[1], Jean-Marie Burel[1], Erin Diel[2], David Gault[1], Kevin Kozlowski[2], Dominik Lindner[1], Melissa Linkert[2], Trevor Manz[3], Will Moore[1], Constantin Pape[4], Christian Tischer[4], Jason R. Swedlow[1,2]*

[1]University of Dundee, Dundee, Scotland, UK
[2]Glencoe Software, Inc. Seattle, WA, USA
[3]Harvard Medical School, Boston, MA, USA
[4]European Molecular Biology Laboratory (EMBL), Heidelberg, Germany

*email: j.r.swedlow@dundee.ac.uk

Table of Contents

Supplementary Table 1: Characteristics of three binary containers for imaging data……………………………….S-1
Supplementary Note: Scaling strategies for interoperable bioimaging data formats………………………………..S-2
References …….....S-5

Supplementary Table 1: Characteristics of three binary containers for imaging data

	TIFF	HDF5	NGFF
First release	1986	1998	2016
Maturity (in imaging)	Ubiquitous	Well-supported	Emerging
Base structure	Sequence of 2D planes	Hierarchy of ND arrays	Hierarchy of ND arrays
Multi-file support	With OME metadata	With internal links	Natively
Pyramidal images	With OME metadata	BDV, Imaris	OME-Zarr
Advantages	Tool support	Feature rich format	Simplicity
Limitations	Scalability	Parallel writes	Large number of small files
Ideal use case	Laptop	Powerful workstation, or cluster	Online archive, or public resource

Supplementary Note: Scaling strategies for interoperable bioimaging data formats

Introduction

For 15 years, the Open Microscopy Environment (OME) has provided the community an alternative to a common file format, namely the Bio-Formats translation library [1]. Rather than declaring and maintaining a single standardized format, OME's strategy has been to focus on bioimage data access API. This has achieved a *de facto* standardisation of bioimaging metadata, with the OME Data Model [2] being incorporated into many open and commercial software tools and most recently, several "minimal information" specifications across a wide variety of bioimaging domains and applications [3–6]. The diversity of the domains covered by these specifications is an example of the range of applications of biological imaging and pace of innovation discussed in the main text and helps explain why biological imaging has so far failed to agree on and adopt a single data format.

However, the authors acknowledge that no single format is likely to satisfy all use cases, programming environments and technology domains (see Supplementary Table 1 for comparison of functionality and limitations). For instance, the requirements of an imaging technology manufacturer, that needs a file format with optimised write performance, are very different from those of a computational scientist building new machine learning (ML) technologies or a public data repository that has to publish and serve many millions to billions of image datasets. The analysis below details OME's previous experiences trying to bridge this tension and, along with the results reported in the main text, underscores the need for a new chunked, parallelized data storage and access mechanism, enabling advanced AI applications and public data resources.

Limits of On-the-fly Translation

One classically successful approach to provide unified access to bioimaging data uses a library that dynamically translates bioimage metadata and binary data from the innumerable proprietary file formats (PFFs) that exist in this domain (Extended Data Figure 3). This strategy is provided by several open source solutions, including the authors' Bio-Formats [1], a Java-based library that supports >150 PFFs; OpenSlide [7], a C++ library that focuses on PFFs used in whole slide imaging (WSI); and aicsimageio [8], a Python library that wraps vendor libraries for simplified numpy access. Translating data in real-time has worked well for many applications, and such translation libraries have become the reference APIs in their fields. For datasets smaller than 10 gigabytes (GB) that are accessed by a single or a small number of users, this strategy will likely continue.

However, large, public data resources like the Allen Cell Explorer (https://www.allencell.org/), Image Data Resource (IDR; https://idr.openmicroscopy.org) [9], Systems Science of Biological Dynamics Database (SSBD; https://ssbd.riken.jp/database/) [10], and others have revealed the fundamental bottlenecks created by the computational cost and time required for repeated translation of massive collections of PFFs. The same problem occurs in data hungry applications like machine learning, where the overhead of real-time translation precludes the use of even larger, more richly annotated datasets. The common thread in these use cases is high levels of data re-use, where the same binary data may be accessed 1000s of times or more. In this case, repeated translation wastes CPU cycles, access time and energy.

Furthermore, the outputs of these applications remain fundamentally isolated from the original data when either the complexity or licensing of PFFs prevents the writing of further analytical metadata in the same format. This is important because maintaining continuity between original and derived datasets is a key requirement for experimental provenance and reproducibility

(https://www.force11.org/group/fairgroup/fairprinciples). Finally, individual dataset volumes have grown with the advent and popularization of imaging modalities that support large tissue samples, such as digital pathology, light sheet microscopy (LSM), and focused ion beam-scanning electron microscopy. In these applications, efficient, high performance data access requires multi-resolution representations (often referred to as pyramidal data) that enable zoomable visualization and selectable levels of resolution for interactive navigation and scalable analysis. Providing multi-resolution support across >150 PFFs is simply not practical nor computationally efficient. In short, for the applications that are becoming strategic opportunities for new directions in bioimaging, real-time translation no longer scales.

Challenges of Permanent Conversion

A distinct approach -- converting data from PFFs to a common, well-defined format -- solves the computational demands of repeated and real-time image translation but requires a format that has broad application and utility, long-term stability, multiple open-source implementations and the support of the community. Some success has been achieved with OME-TIFF, a 2D-optimized, multi-resolution image format that captures acquisition metadata as OME-XML in the TIFF header [1,2,11]. Reference software implementations are available in Java (https://github.com/ome/bioformats/), C++ (https://gitlab.com/codelibre/ome/ome-files-cpp) and Python (e.g., https://github.com/AllenCellModeling/aicsimageio, https://github.com/apeer-micro/apeer-ometiff-library, https://github.com/cgohlke/tifffile). OME-TIFF is supported by several commercial imaging companies (see https://www.openmicroscopy.org/commercial-partners/) and is the recommended format for public data projects like Image Data Resource (IDR) or Allen Institute of Cell Science, making their data available from https://open.quiltdata.com/b/allencell/.

As our and others' use of existing tools for conversion to OME-TIFF grew, TIFF's linear binary layout became a bottleneck. Larger files took increasingly long to write. This problem was most obvious in projects that required the conversion of large numbers of whole slide images from PFFs to OME-TIFF for use in "data lakes", large repositories of structured and unstructured data that are used for AI training sets (https://pathlake.org; https://icaird.com). The need for a scalable conversion motivated our development of two tools, *bioformats2raw (https://github.com/glencoesoftware/bioformats2raw)* and *raw2ometiff (https://github.com/glencoesoftware/raw2ometiff)*. Together they provide a parallel pipeline using Bio-Formats to convert any supported PFF into multi-resolution OME-TIFF. This is achieved by breaking images into atomic "chunks", writing them independently to disk, and generating pyramidal data from them when none are available, whereupon a second process can efficiently write these chunks into TIFF (Extended Data Figure 3). With this conversion pipeline, OME-TIFF becomes a performant solution for domains handling larger planar data, for example whole slide images in digital pathology.

A fundamental issue with OME-TIFF, however, is that though it supports 5D images (e.g., space, time, channel), its binary data access remains limited to TIFF's 2D tiles. This means that loading a small 3D region requires multiple reads from different locations in a file rather than reading one contiguous block. While storage of individual planes of image data that encompass hundreds of GB and dozens of pyramidal resolutions of data is possible, performance suffers when scaling to multi-dimensional, multi-TB datasets, e.g., LSM datasets. The benchmark we report in the main text of this article is an example, where the LSM-like test data formatted in TIFF consumed too much memory for completing the benchmark.

One solution to the dimensionality issue is HDF5, a multi-dimensional data format that internally supports chunk-based access [12]. Several open HDF5 bioimage file formats have been designed and implemented [13–16] and libraries exist for these various formats in several of the major programming languages. The HDF5-based BigDataViewer file format [14] has proven to be quite powerful for the LSM community, as it provides a convenient integrated format and the chunking required for interactive visualization of the large 3D timelapse datasets produced by LSM. Oxford Instruments have released another open HDF5-based implementation that is widely used and includes a format specification

(https://imaris.oxinst.com/support/imaris-file-format) as well as an open reference implementation, ImarisWriter [17].

These formats have performed well for interactive visualization and analysis, but there remain limitations for processing and integration of large numbers of HDF5 datasets. Parallel writing of HDF5 files is not supported for regular users without specialised environments. Server applications like HSDS mitigate this problem, but require additional dependencies and computational power, making solutions harder to adopt and thus less widely available to the global community. Also, unlike TIFF, the HDF5 format does not inherently specify an N-dimensional image type, so each HDF5 bioimaging implementation is in effect its own file format, growing the number of PFFs.

An issue with both TIFF and HDF5 is that any format must eventually be stored on a hard-drive or other permanent memory structure. Filesystems have been the workhorse of the imaging community since its inception. They enable low-latency, "random access" to large binary data files. This speed is an underlying assumption of most visualization and analysis applications, but filesystems are relatively expensive and their complexity comes with relatively high maintenance costs. With the ascendance of cloud computing, an alternative is to use a loosely-defined storage technology (often called "object storage") that treats individual files as distinct immutable objects. Object storage provides relatively simple read and write procedures that transfer whole objects (often called "chunks"). Each object is stored redundantly across multiple servers, which offers improved parallelization and scalability in exchange for increased access latency. To make use of the advantages of object storage, a modern format, accommodating contemporary dataset volumes and dimensionality, is needed that does not require the whole binary structure to be accessed as a single monolithic block on disk or in the cloud. While a monolithic strategy works for smaller files, it is fundamentally limiting for multi-TB, multi-dimensional and multi-modal imaging datasets.

While the TIFF and HDF5 implementations remain valuable in their respective domains, their inability to cover all use cases are examples of the pitfalls of format standardization. Creating a data format standard requires equal consideration for performance, usability, and structure, with a balance of community-driven specification and extensibility. Historically, OME-TIFF and OME-XML were highly specified but lacked optimal adaptability to novel data volumes and high dimensionality, while HDF5 was highly extensible and thereby suffered a branching into multiple PFFs. Improved performance motivated early adoption, especially given high usability, but providing a clear structure for binary data and metadata is essential to yield a cohesive landscape of new tools, rather than a divergence of format variants. Extensibility, however, is necessary for adoption by new domains or vendors and integration with novel analytical approaches that were not considered at the point of initial specification.

Formats with Interoperable Metadata

Our work is based on the premise that a single file format cannot be optimal for the wide variety of bioimaging use cases. How can we build and use multiple open bioimaging formats without again requiring a translation library? The solution is to guarantee interoperability between the formats themselves. All data in one format should be losslessly convertible to the other, supported formats. The key to achieving this is a re-evaluation of the metadata format that OME has been maintaining for over 15 years. The Zarr format described in the main text provides the opportunity for this. Zarr metadata is stored as a block of JSON at each level of a hierarchy of arrays. This framework is simple enough to be represented in HDF5's more powerful attribute system, but also powerful to encompass OME-TIFF metadata completely (Extended Data Figure 4).

Additionally, this redefinition of the OME metadata for NGFFs provides an opportunity to specify conventions that will apply to and support the breadth of formats needed by the community. In collaboration with RIKEN, an initial version of a Resource Description Framework (RDF)-based representation of OME metadata has been published [18,19]. We aim to store this RDF metadata in its JSON-LD representation within the NGFF

structure, permitting third-party definition of metadata specifications as well as integration with existing efforts to improve the findability of datasets like Bioschemas [20].

While the rapid pace of innovation in bioimaging continues, the core data structures -- binary pixel data and experimental, acquisition and analytic metadata -- are likely common to most imaging experiments and datasets. Indeed, recent work on minimal bioimaging metadata standards have focused on a collection of core concepts including those from OME that can be extended to support domain- and application-specific requirements [4,21] . As a participant in the “Recommended Metadata for Biological Images” effort to develop a minimal bioimaging metadata specification [3], we aim to provide open reference software and formats that implement the proposed minimal metadata specification and can be widely adopted and extended by the bioimaging community.

Building a Community Format

As we investigated candidates for the intermediate multifile, "chunked" representation for parallelizing the *bioformats2raw/raw2ometiff* conversion pipeline, we realized that these data structures could complement existing TIFF- and HDF5-based workflows and enable new types of parallel bioimaging use cases. We collectively refer to these formats as NGFFs. We have focused on two very similar, open source strategies for laying out binary data. N5 (https://github.com/saalfeldlab/n5) is a binary data format that uses embedded directories and defined multi-dimensional chunking to provide fast, cloud-competent image data storage. N5 was developed by the Fiji community [22] and there are now several examples of public datasets in N5. Zarr (https://github.com/zarr-developers) follows a nearly identical strategy of storing chunks in individual files across directories and was originally adopted for handling genomic and geospatial data, similarly with a number of datasets available publicly [23]. Since 2018, the two communities have worked together to unify the two formats via an updated Zarr specification (v3). That work forms the basis for OME-NGFF as reported in the main article.

It should be noted that although these formats provide an initial basis for development, as the needs of the community are catalogued, more sophisticated formats like TileDB Embedded may join this list of supported NGFF formats. TileDB Embedded uses a database-like storage engine based on sparse and dense multi-dimensional arrays to service chunks of data and as it is an open source C++ run-time library, data can be accessed from Java, Python and several other programming languages. However, a long-term commitment to format support by the users, developers, and funders should be demonstrated to avoid a new translation bottleneck.

In summary, we hope to show that by covering the breadth of community requirements with an interoperable suite of select formats we can ultimately deliver a truly FAIR bioimaging data standard.

References

1. Linkert, M. *et al.* Metadata matters: access to image data in the real world. *J. Cell Biol.* **189**, 777–782 (2010).
2. Goldberg, I. G. *et al.* The Open Microscopy Environment (OME) Data Model and XML file: open tools for informatics and quantitative analysis in biological imaging. *Genome Biol.* **6**, R47 (2005).
3. Sarkans, U. *et al.* REMBI: Recommended Metadata for Biological Images-enabling reuse of microscopy data in biology. *Nat. Methods* (2021) doi:10.1038/s41592-021-01166-8.
4. Ropelewski, A. J. *et al.* Essential Metadata for 3D BRAIN Microscopy. *arXiv [q-bio.OT]* (2021).
5. Wittner, R. *et al.* ISO 23494: Biotechnology – Provenance Information Model for Biological Specimen And Data. in *Provenance and Annotation of Data and Processes* 222–225 (Springer International Publishing, 2021). doi:10.1007/978-3-030-80960-7_16.
6. Holub, P. *et al. Towards a Common Standard for Data and Specimen Provenance in Life Sciences*. (2021).

doi:10.5281/zenodo.5093125.
7. Goode, A., Gilbert, B., Harkes, J., Jukic, D. & Satyanarayanan, M. OpenSlide: A vendor-neutral software foundation for digital pathology. *J. Pathol. Inform.* **4**, (2013).
8. AICSImageIO Contributors. *AICSImageIO: Image Reading, Metadata Conversion, and Image Writing for Microscopy Images in Pure Python*. (GitHub).
9. Williams, E. *et al.* The Image Data Resource: A Bioimage Data Integration and Publication Platform. *Nat. Methods* **14**, 775–781 (2017).
10. Tohsato, Y., Ho, K., Kyoda, K. & Onami, S. SSBD: a database of quantitative data of spatiotemporal dynamics of biological phenomena. *Bioinformatics* (2016).
11. Besson, S. *et al.* Bringing Open Data to Whole Slide Imaging. in *Digital Pathology* 3–10 (Springer International Publishing, 2019). doi:10.1007/978-3-030-23937-4_1.
12. The HDF Group. *Hierarchical Data Format*. (1997-2021).
13. Sommer, C., Held, M., Fischer, B., Huber, W. & Gerlich, D. W. CellH5: a format for data exchange in high-content screening. *Bioinformatics* **29**, 1580–1582 (2013).
14. Pietzsch, T., Saalfeld, S., Preibisch, S. & Tomancak, P. BigDataViewer: visualization and processing for large image data sets. *Nat. Methods* **12**, 481–483 (2015).
15. Ingargiola, A., Laurence, T., Boutelle, R., Weiss, S. & Michalet, X. Photon-HDF5: An Open File Format for Timestamp-Based Single-Molecule Fluorescence Experiments. *Biophys. J.* **110**, 26–33 (2016).
16. Millard, B. L., Niepel, M., Menden, M. P., Muhlich, J. L. & Sorger, P. K. Adaptive informatics for multifactorial and high-content biological data. *Nat. Methods* **8**, 487–493 (2011).
17. Beati, I., Andreica, E. & Majer, P. ImarisWriter: Open Source Software for Storage of Large Images in Blockwise Multi-Resolution Format. *arXiv [cs.DC]* (2020).
18. Kobayashi, N., Moore, J., Onami, S. & Swedlow, J. R. OME Core Ontology: An OWL-based Life Science Imaging Data Model. in 149–150.
19. Moore J, Kobayashi N, Kunis S, Onami S, Swedlow JR. On Bringing Bioimaging Data into the Open (World). in *SWAT4HCLS 2019* 44–53.
20. Gray, A., Goble, C. & Jimenez, R. Bioschemas: From Potato Salad to Protein Annotation. https://iswc2017.semanticweb.org/paper-579/.
21. Nelson, G. *et al.* QUAREP-LiMi: A community-driven initiative to establish guidelines for quality assessment and reproducibility for instruments and images in light microscopy. *arXiv [q-bio.OT]* (2021).
22. Schindelin, J. *et al.* Fiji: an open-source platform for biological-image analysis. *Nat. Methods* **9**, 676–682 (2012).
23. Miles, A. *et al. zarr-developers/zarr-python: v2.5.0*. (2020). doi:10.5281/zenodo.4069231.
